# Magnetic resonance imaging based radiomics for predicting pathogenetic features and survival in rectal cancer

**DOI:** 10.3389/pore.2026.1612303

**Published:** 2026-05-07

**Authors:** Bertalan Toth, Marcell Mesterházi, Istvan Szabo, Tamas Mersich, Erika Toth, Janos Szoke, Andras Benczur, Csaba Kerepesi, David Laszlo Tarnoki, Adam Domonkos Tarnoki

**Affiliations:** 1 Faculty of Medicine, Semmelweis University, Budapest, Hungary; 2 HUN-REN Institute for Computer Science and Control, Budapest, Hungary; 3 Oncologic Imaging and Invasive Diagnostic Centre and the National Tumor Biology Laboratory, National Institute of Oncology, Budapest, Hungary; 4 Department of Surgery, National Tumor Biology Laboratory, National Institute of Oncology, Budapest, Hungary; 5 Department of Pathology, National Tumor Biology Laboratory, National Institute of Oncology, Budapest, Hungary

**Keywords:** colorectal neoplasms, magnetic resonance imaging, mutation, prognosis, radiomics

## Abstract

**Background:**

Prediction of pathologic features and outcome in patients with rectal cancer is challenging as a result of lack of a significant biomarker and heterogeneity between and within tumors. This study aims to evaluate the potential of Magnetic Resonance Imaging (MRI)-based radiomics in predicting key pathological features and long-term survival outcomes in patients.

**Methods:**

A retrospective study was conducted on 510 rectal cancer patients treated between 2015 and 2019. The inclusion criteria required pre-therapeutic MRI performed on a Discovery MR750W 3.0T machine and known KRAS mutation status. Forty-seven patients met the criteria. MRI sequences included T1-weighted, T2-weighted fat-saturated (T2FS), high-resolution T2-weighted (T2HR), and diffusion-weighted imaging (DWI). Radiomic features were extracted using PyRadiomics, and machine learning models were developed using XGBoost and LightGBM classifiers. Feature selection was performed using Sequential Feature Selector (SFS) and Minimum Redundancy Feature Selection (mRMR).

**Results:**

The model for KRAS mutation status achieved an Area Under the ROC curve (AUC) of 0.7475 (training) and 0.75 (testing). Lymph node invasion prediction had an AUC of 0.7892 (training) and 0.7984 (testing). Vascular invasion prediction yielded an AUC of 0.6989 (training) and 0.7143 (testing). The 5-year survival prediction model showed an AUC of 0.7848 (training) and 0.7750 (testing). Metastasis prediction achieved an AUC of 0.6627 (training) and 0.6857 (testing).

**Conclusion:**

MRI-based radiomics demonstrates significant potential in predicting key pathological features and long-term survival outcomes in rectal cancer patients. Integrating multimodal imaging data and clinical information, along with automated segmentation techniques, could further enhance model accuracy and clinical utility.

## Introduction

Cancers of the colon and rectum affect one in every ten cancer patients, making it the third most common type of cancer worldwide and the second leading cause of cancer-related deaths [1]. By 2035, the incidence is projected to exceed 2.5 million new cases annually [2]. The overall prognosis of colorectal cancer (CRC) patients is influenced by several factors, most of which are determined through invasive methods, mostly pathological examination of the removed specimen.

Rectal cancer and its treatment can significantly impact patients’ quality of life, affecting anal continence, sexual function and urinary function. However, life expectancy remains relatively high, with a 5-year overall survival rate exceeding 84%. While a radical surgical approach whether open or minimally invasive is crucial for long-term survival, it is also a primary contributor to reduced quality of life. Recent advancements in neoadjuvant therapy have introduced total neoadjuvant therapy (TNT) as a promising strategy to achieve complete clinical and pathological response, even in patients with locally advanced rectal cancer [3].

Avoiding radical surgery has led to the adoption of a “watch-and-wait” strategy. However, the absence of total mesorectal excision (TME) and the limited tissue obtained through local excision make thorough clinical evaluation of the pelvis essential [4].

Traditional prognostic factors such as lymph node invasion, tumor regression grade, tumor budding, and extramural venous invasion (EMVI) are assessed using MRI at various treatment stages. However, these factors are typically confirmed through pathological evaluation of resected specimens. The discrepancy between MRI findings and the pathological characteristics of non-resected tumors is a critical challenge in decision-making, particularly in optimizing the timing of salvage surgery when following a watch-and-wait protocol. Radiomic analysis, in addition to conventional MRI evaluation, offers a novel perspective on rectal cancer prognosis. By providing a more precise correlation between imaging features and both traditional and emerging prognostic factors such as lymphatic and venous invasion or metastatic potential radiomics enhances the accuracy of risk stratification and long-term outcome prediction [5].

Another significant prognostic factor is the presence of Kristen rat sarcoma (KRAS) mutations, found in 35%–40% of CRC cases. Targeted therapies, such as epidermal growth factor receptor inhibitors, are used in CRC treatment but are significantly less effective in the presence of KRAS mutations [6, 7]. The National Comprehensive Cancer Network recommends cetuximab or panitumumab for late-stage CRC patients, making it essential to determine the mutational status of these patients [8]. Currently, KRAS examination requires histopathological samples typically obtained via colonoscopy. However, in advanced cases, limited mobility of the camera can result in insufficient samples for determining KRAS status.

In this study, we also investigated four pretherapeutic MRI sequences to identify significant radiomic features that could predict various pathological characteristics, such as KRAS mutation, metastasis, lymph-node invasion, vascular invasion, and five-year prognosis on the same patient population. Then with the help of machine learning and classification algorithms integrating the radiomic features and additional patient data we aimed to build a model to be used in a clinical setting and for personalized medicine.

## Materials and methods

The research (“*Radiomic Analysis of Breast Tumors, Colorectal, Head and Neck, and Ovarian Cancers*”) has been ethically approved by the Regional Research Ethics Committee and the Scientific and Research Ethics Committee of the Health Science Council (07464-3/2023/EÜIG). All methods were carried out in accordance with relevant guidelines and regulations. This study was reported in accordance with the main domains of the CLEAR radiomics checklist. However, not all items could be fully addressed, particularly uncertainty measures, and open science items, which we acknowledge as limitations. Due to the retrospective nature of the study, National Ethical Review Board waived the need of obtaining informed consent. This retrospective study enrolled 510 patients who underwent treatment for rectal cancer at the National Institute of Oncology, Budapest, Hungary, between 2015 and 2019. The inclusion criteria for the study were to ensure consistency and reliability of the data. Firstly, patients must have undergone surgery at our institution with proven colorectal adenocarcinoma. Secondly, the MRI must have been performed on our designated 3T MRI machine prior to any therapeutic intervention, ensuring that the imaging data was consistent and comparable across all patients. Lastly, the KRAS mutation status of the patients had to be known, and all additional relevant clinical and pathological information had to be accessible through the institution’s archive. Finally, 47 patients were included in the study with a mean age of 59.72 years (SD 8.959) at the time of surgery (Table 1).

**TABLE 1:** The characteristics table of the patient cohort.

Characteristics	Patients	Percentage
​	47	​
Age	59.72 (SD 8.959)	​
Gender		
Male	25	53.2%
Female	22	46.8%
Grade		
1	33	70.2%
2	9	19.2%
3	5	10.6%
pT stage		
T1	3	6.4%
T2	11	23.4%
T3	25	53.2%
T4	8	17.0%
pN stage		
N0	20	42.6%
N1	20	42.6%
N2	6	12.8%
KRAS mutation status		
Mutated	30	63.8%
Wild	17	36.2%
Metastasis		
Positive	29	61.7%
Negative	18	38.3%
Vascular invasion		
Positive	22	46.8%
Negative	25	53.2%
Neural invasion		
Positive	7	14.9%
Negative	40	85.1%

Clinical characteristics such as age, gender, smoking, alcohol intake, TNM status, pathological features, and surgical method were collected for each patient. The metastatic status of the patients encompassed both synchronous and metachronous metastases based on the available documentation.

### MRI

The MRI acquisition for this study was conducted using the Discovery MR750W 3.0T machine, specifically for staging examinations of rectal cancer patients prior to any therapeutic intervention. These patients had previously undergone a positive colonoscopy confirming adenocarcinoma. The MRI scans focused exclusively on the pelvic region using the rectal MRI protocol of our institution. The detailed acquisition parameters can be found in Table 2. Four sequences were utilized in the acquisition process: T1-weighted, T2-weighted fat-saturated, high-resolution T2-weighted (T2HR), and diffusion-weighted imaging (DWI) at the lowest b value. This approach was necessary to provide high-quality, detailed images for accurately defining the region of interest (ROI) and delineating the tumor borders from the surrounding tissue. Such precision in imaging was a must for the accurate staging and assessment of rectal cancer, contributing significantly to the study’s overall objective.

**TABLE 2:** Containing the rectal protocol acquisition parameters.

MRI acquisition parameters	T1	T2FS	T2 HR	DWI
RT	689	9850	7200	8300
ET	21	83	97	53
ST	5 mm	5 mm	3.6 mm	3.6 mm
Resolution	512 × 512/0.6 × 0.6 × 0.5 mm^3^	512 × 512/0.6 × 0.6 × 0.5 mm^3^	512 × 512/0.4 × 0.4 × 3 mm^3^	256 × 256/1.5 × 1.5 × 3 mm^3^
ETL	41	7	6	​
Vendor coil	Ge medical system body 36 AA2			
b-values	​	​	​	50, 800, 1400

### Pathology and genetic oncology

During the course of the therapy, patients who were eligible underwent surgery to remove the tumor and surrounding tissue, including the lymph nodes. The excised specimens were then sent to the pathology department for detailed examination. The pathological analysis involved a thorough histopathological evaluation of the tumor and the surrounding tissues to assess the extent of cancer invasion and the involvement of lymph nodes. The information obtained from these examinations included the tumor grade, pathological TNM staging, vascular invasion, perineural invasion, invasion of lymph nodes, and the response to neoadjuvant radiotherapy. For genetic testing, we used the COBAS KRAS Mutation Test v2 Kit, which is designed to detect mutations in exons 2, 3, and 4 of the KRAS gene in formalin-fixed, paraffin-embedded (FFPE) tissue samples obtained through biopsy during the course of the therapy.

### Segmentation

**FIGURE 1:**
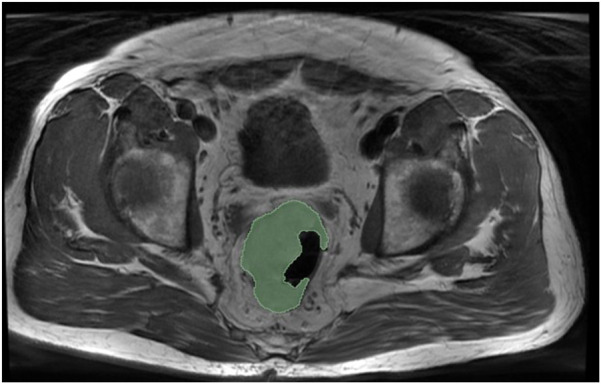
Axial T1-weighted MRI sequence of the pelvic region in a colorectal cancer patient. The green area indicates the segmented tumor region of interest (ROI), from which radiomics parameters were computed. This segmentation method was then applied across all MRI sequences used in the study.

The segmentation of the MR images was performed using the 3D Slicer program, a versatile and widely used open-source software platform for medical image informatics, image processing, and three-dimensional visualization [9]. Initially, all patient information was anonymized, and each patient was assigned a unique ID to maintain confidentiality and ensure data integrity. The ROI was precisely segmented for each patient and each MRI sequence. To ensure homogeneity and consistency in the segmentation process, a single operator conducted all segmentations. The operator aimed to include the entire tumor in the segmentation mask. This approach ensured comprehensive and accurate delineation of the tumor boundaries, which was crucial for subsequent radiomic feature extraction and analysis (Figure 1).

### Feature extraction

All 3D image volumes and corresponding segmentations were resampled to an isotropic spatial resolution of 1 × 1 × 1 mm^3^ prior to radiomics feature extraction. Intensity values were subsequently scaled and normalized to reduce inter-scan variability. The program used for feature extraction in this study was PyRadiomics, an open-source python package designed for the extraction of a large number of radiomic features. The default settings are in line with Image Biomarker Standardisation Initiative (IBSI) guidelines for feature definitions and nomenclature, but minor deviations exist that can be found in the PyRadiomics documentation. PyRadiomics is widely recognized for its flexibility in handling various imaging modalities and providing comprehensive radiomic analyses. From the segmented MR images, a total of 1241 radiomic features were extracted. These features are categorized into several groups, including first-order statistics, shape-based features, and texture features [10].

First-order statistics describe the distribution of voxel intensities within the ROI, providing basic information about the tissue’s intensity values. Shape-based features capture the geometric properties of the tumor, such as volume, surface area, and sphericity, which are for understanding the tumor’s physical characteristics. Texture features, on the other hand, describe the spatial arrangement and relationship of pixel intensities within the ROI, revealing patterns and structures that might not be apparent through visual inspection alone. These texture features are further divided into subcategories, such as gray-level co-occurrence matrix (GLCM), gray-level run length matrix (GLRLM), gray-level size zone matrix (GLSZM), neighboring gray-tone difference matrix (NGTDM), and gray-level dependence matrix (GLDM), each providing unique insights into the tissue architecture and heterogeneity. By analyzing these diverse radiomic features, the study aimed to uncover significant correlations between MRI characteristics and pathological and molecular tumor attributes [11].

### Feature selection and model creation

The previously computed radiomic features were merged with the patient information we collected earlier. Our task was divided by five target labels: (a) KRAS mutation status, (b) lymph node invasion, (c) metastasis, (d) vascular invasion, and (e) 5-year survival. The dataset for each target task was then split into a training group and a test group in a 75:25 ratio, maintaining the original ratio of the target label in each different task (e.g., if 25% of the patients were KRAS-mutated in the whole dataset, the training and test groups also had 25% KRAS-mutated patients). The clinical covariates included age, gender, diabetes status, smoking status, and metastasis. For the model in which metastasis was the target variable, this covariate was excluded from the input features.

In the training group, we employed two types of feature selection methods: Sequential Feature Selector (SFS) [12] and Minimum Redundancy Feature Selection (mRMR) [13]. SFS is an iterative method that adds or removes features based on a performance criterion, ensuring that the selected features contribute the most to the model’s accuracy. The mRMR method selects features that are highly relevant to the target variable while being minimally redundant to each other, thus optimizing the feature set for maximum information gain with minimal redundancy.

For model creation, we used the XGBoost Classifier and LightGBM Classifier. XGBoost (Extreme Gradient Boosting) is a powerful, scalable machine learning system for tree boosting that uses a gradient boosting framework. It is known for its efficiency, accuracy, and support for parallel computation [14]. LightGBM (Light Gradient Boosting Machine) is another high-performance gradient boosting framework that is particularly optimized for speed and efficiency with large datasets. It uses a histogram-based algorithm to speed up training [15].

We optimized the hyperparameters of these models to boost their performance. The features that provided the best Area Under the ROC Curve (AUC) values during training were selected for each target label. We then identified the best combination of features and models that provided the highest AUC scores with low standard deviation on the training group.

In addition, confusion matrices were computed and standard performance metrics including sensitivity, specificity, positive predictive value (PPV), and negative predictive value (NPV) were calculated (Table 3).

**TABLE 3:** Confusion Matrix, Sensitivity, Specificity, Positive Predictive value, Negative Predictive value for each target model.

Model	Mutation	Lymphnode invasion	Vascular invasion	5 years survival	Metastasis
Confusion matrix	[[23.7][12.4]]	[[12.10][3.20]]	[[16.12][0.19]]	[[7.8][4.28]]	[[12.8][0.28]]
Sensitivity	0.250	0.870	1.000	0.875	1.000
Specificity	0.767	0.545	0.571	0.467	0.600
PPV	0.364	0.667	0.613	0.778	0.778
NPV	0.657	0.800	1.000	0.636	1.000

Each model was then evaluated on the test group, and we generated ROC-AUC diagrams for each target group. Additionally, we created a table listing the features that were important for each model, providing insight into the factors most strongly associated with each target outcome.

## Results

**FIGURE 2:**
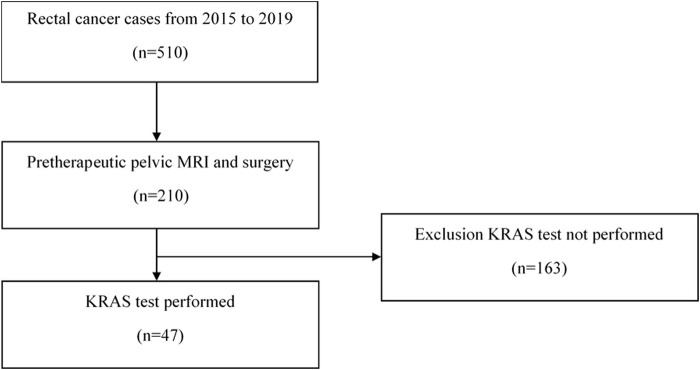
The Inclusion/Exclusion criteria flowchart of the cohort group.

After applying the inclusion and exclusion criteria outlined in the methods (Figure 2), the study cohort consisted of 47 patients. The cohort had a mean age of 59.72 ± 8.959 years with 25 men and 22 women. The predictive performance of the models was evaluated for various clinical outcomes, with the results summarized in Table 4. The AUC values for each model were assessed during both the training and test phases.

**TABLE 4:** The training and test results of the 5 models based on the 5 target labels.

Model	Training AUC	Standard deviation	Test AUC
KRAS mutation status	0.7475	0.0775	0.7500
Lymph node invasion	0.7892	0.1670	0.7984
Vascular invasion	0.6989	0.0073	0.7143
5-Year survival	0.7848	0.0459	0.7750
Metastasis	0.6627	0.0421	0.6857

**FIGURE 3:**
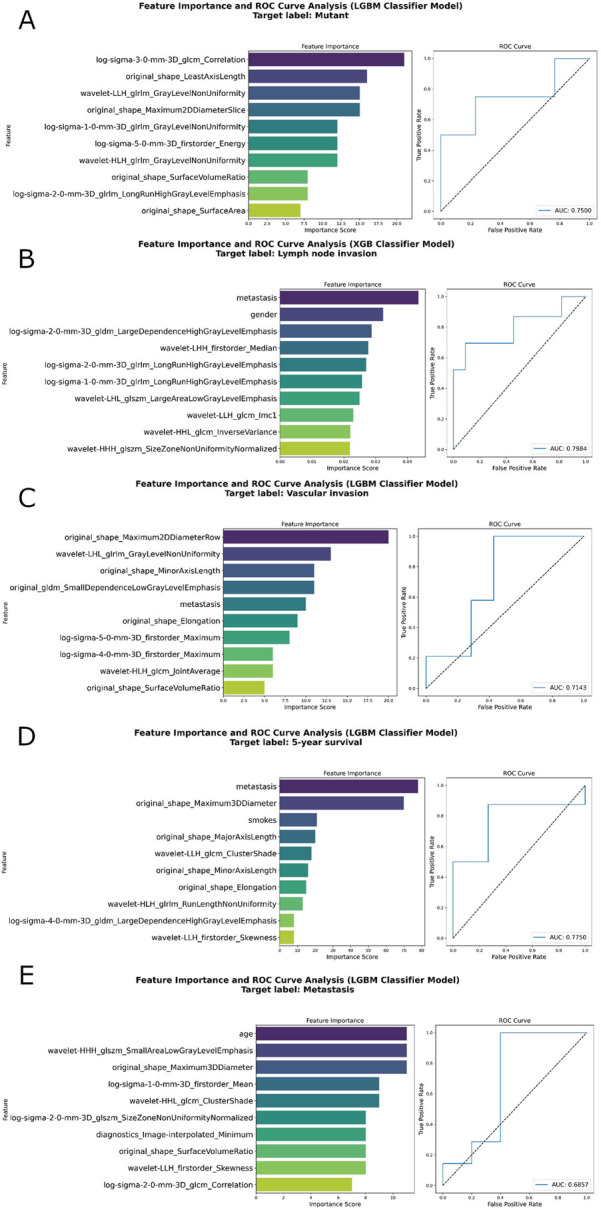
Feature importance and ROC curve for the prediction models. The left panel displays the top ten most important radiomic features ranked by their importance scores and the right panel shows the Receiver Operating Characteristic (ROC) curve, with the Area Under the Curve (AUC) values on the test data set, indicating the model’s predictive performance. The target labels for the study are KRAS mutation status **(A)**, lymph node invasion **(B)**, vascular invasion **(C)**, 5-year prognosis **(D)**, and the metastasis **(E)**.

For the KRAS mutation status model, the AUC was 0.7475 during training and 0.7500 in testing (Figure 3A). The lymph node invasion model exhibited a training AUC of 0.7892 and a test AUC of 0.7984 (Figure 3B), indicating strong predictive ability. The vascular invasion model showed a more modest performance, with training and test AUCs of 0.6989 and 0.7143, respectively (Figure 3C). The 5-year survival model demonstrated consistent results, with a training AUC of 0.7848 and a test AUC of 0.7750 (Figure 3D). Finally, the metastasis prediction model showed the lowest performance, with training and test AUCs of 0.6627 and 0.6857, respectively (Figure 3E).

**FIGURE 4:**
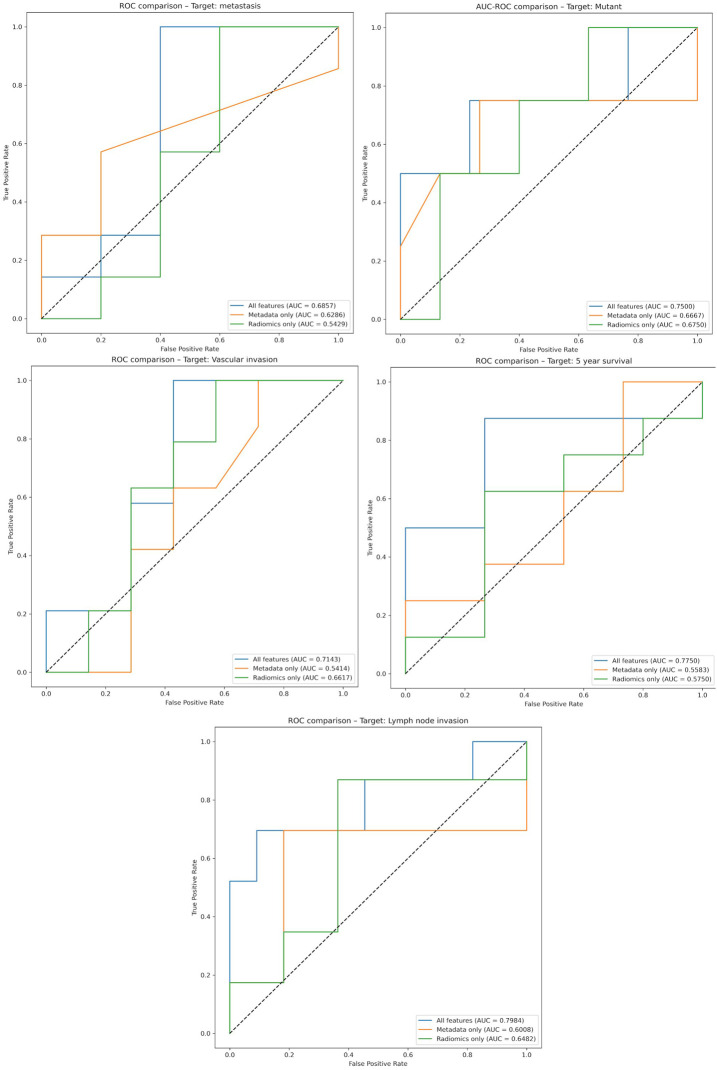
ROC curves of the target labels based on 3 separate models, one using only clinical variables, one using only radiomic features, and one combining both.

To avoid overestimating model performance, we trained and evaluated three separate models: one using only clinical variables, one using only radiomic features, and one combining both. The corresponding ROC curves and AUC values are shown, allowing direct comparison of the added value of radiomics beyond clinical variables (Figure 4).

In addition to developing a predictive model for lymph node invasion in rectal cancer, we also calculated the AUC for MRI-based predictions made by a radiologist on the same dataset. The model achieved an AUC of 0.7984, demonstrating a strong ability to distinguish between cases with and without lymph node invasion. In comparison, the radiologist’s assessment on MRI images yielded an AUC of 0.609, indicating a notably lower discriminatory power.

## Discussion

In this study, we used MRI based radiomic to predict key pathological features in rectal cancer focusing on KRAS mutation status, lymph node invasion, metastasis and 5-year survival. Our models demonstrated varying levels of predictive performance, with the lymph node invasion model showing the highest test AUC of 0.7892, followed by the KRAS mutation status model (AUC = 0.75). The models for vascular invasion and metastasis showed more modest results (AUC = 7143 and 0.6857, respectively), while the 5-year survival model had a promising AUC of 0.7750.

Compared with existing literature, our findings are generally consistent with previous studies. To our knowledge, this is the first study to examine five distinct target groups using MRI radiomics in the same cohort of rectal cancer patients. The methodology is also unique in that we utilized data from four different MRI sequences simultaneously for model development, combining multiple feature selection algorithms. Several previous publications explored the association of MRI radiomics and genetic makeup of tumors [16, 17].

For KRAS mutation status, our model achieved an AUC of 0.7475, which aligns well with studies by Alshuhri et al., who reported an AUC of 0.68–0.71 using T2-weighted fat-saturated and diffusion-weighted MRI images, and Cui et al., who achieved an AUC of 0.68 in testing dataset with a similar MRI radiomics approach and a with a larger sample size. Alshuhri et al. examined a patient cohort comparable to ours, with a similar sample size of 60 patients and a comparable mean age of 56.3 years. In terms of tumor stage distribution, their cohort included a higher proportion of patients with more advanced disease, as ∼86% were staged T3 or T4 compared to ∼70% in our cohort. Methodologically, they used only a 1.5 T MRI scanner, whereas we employed a 3.0 T system. The use of a higher-field MRI may have allowed more detailed depiction of tumor morphology and an improved signal to noise ratio, which could have contributed to the performance of our radiomics models. These studies emphasize the importance of T2W MRI scans and texture features in accurately predicting KRAS mutations [18, 19].

Our lymph node invasion model’s AUC of 0.7892 is comparable to that reported by Yang et al., who achieved an AUC of 0.87 using multiparametric MRI radiomics. Yang et al.'s approach of using high-resolution imaging and incorporating clinical risk factors into the radiomics model could explain their superior performance. Their study also included roughly twice as many patients as ours, with 139 participants and a similar proportion of lymph node–positive cases of around 50%. Additionally, their focus on multiparametric MRI and the inclusion of both intratumoral and peritumoral features may have enhanced their model’s accuracy and generalizability. This highlights the importance of integrating multiple imaging modalities and clinical data to improve the predictive power of radiomics models [20].

Our study’s model for predicting vascular invasion achieved an AUC of 0.6989 in the training set and 0.7143 in the test set, using texture and shape features from T1, T2FS, T2HR, and DWI sequences. In contrast, the study by Zhang et al. employed a multimodal radiomics model integrating PET-CT and clinical factors for preoperative prediction of lymphovascular invasion LVI in CRC and reported a significantly higher AUC of 0.918 (95% CI 0.782, 0.982). This multimodal approach likely captured a broader range of tumor characteristics, leading to improved prediction performance. While our MRI-based model provides valuable insights, integrating additional modalities like PET and CT, along with clinical predictors, could raise the accuracy and reliability of vascular invasion predictions in CRC patient [21].

Our results for predicting both synchronous and metachronous metastasis in CRC patients, achieved and AUC of 0.6627. In comparison, Yao et al.'s study employed multiparametric MRI radiomics, including T2-weighted imaging, DWI, and contrast-enhanced T1-weighted imaging (CE-T1), and reported an AUC of 0.79 for predicting recurrence and metastasis. Their patient cohort was substantially larger, comprising 234 patients from two centers, which allowed the inclusion of an additional external validation cohort. Moreover, the region of interest was selected by two radiologists, thereby achieving significantly better results. Thus, integrating multiple MRI sequences and clinical data to strengthen the accuracy of metastasis prediction models in CRC patients [22].

Our study achieved an AUC of 0.7848 for predicting the 5-year survival of colorectal cancer patients using MRI-based radiomics. In contrast, a multi-center study published in Cancer Imaging developed a radiomics-based model using CT images and a random forest classifier to predict disease-free survival and the benefit of adjuvant chemotherapy in stage II CRC patients. This study achieved significant predictive performance by integrating clinical data and employing advanced statistical methods such as the C-index and Kaplan-Meier plots [23]. Similarly, Lv et al utilized PET/CT radiomics with random survival forest models, demonstrating high accuracy in predicting prognosis by combining multiple imaging features [24].

One of the main constraints is our reliance on single-modality imaging without incorporating additional modalities like CT or PET/CT limit the predictive power of our models. The relatively small sample size and manual segmentation process also pose challenges to generalizability and efficiency. Future studies should focus on integrating multimodal imaging and clinical data, using automated segmentation, and validating findings in larger cohorts to improve the results and applicability of radiomics in rectal cancer prognosis. This could be mitigated in future studies by using automated segmentation techniques.

In conclusion, our study demonstrates that MRI radiomics holds significant potential in predicting key pathological features in rectal cancer patients. In the evolving clinical landscape of total neoadjuvant therapy (TNT) and the watch-and-wait strategy for rectal cancer, pelvic MRI plays a crucial role in guiding management decisions, including salvage surgery and follow-up strategies. As radical surgery is increasingly being replaced by local excision in patients achieving complete clinical response (cCR) or near-complete clinical response (nCR) following various chemotherapy and radiation protocols, the absence of surgically removed tissue limits the pathological assessment of key prognostic factors. This challenge underscores the need for improved MRI interpretation and radiomics-based approaches to provide reliable surrogate markers for tumor response. Given that our lymph node invasion model performed well, MRI radiomics may serve as a critical tool for identifying patients at risk of residual nodal disease, thereby influencing multidisciplinary team (MDT) driven decisions regarding surveillance and adjuvant therapy. Since the optimal sequencing, chemotherapeutic regimens, and timing of TNT are still under investigation, MRI assessments remain variable but increasingly impactful in MDT planning. Further refinement of MRI radiomics could enhance its role in treatment stratification and long-term disease monitoring in rectal cancer patients [25, 26].

## Data Availability

The data analyzed in this study is subject to the following licenses/restrictions: The datasets generated or analyzed during the study are not publicly available due to institutional policies and legal regulations governing data sharing but are available from the corresponding author on reasonable request. Requests to access these datasets should be directed to toth.bertalan00@gmail.com.
